# *E. coli* Group 1 Capsular Polysaccharide Exportation Nanomachinary as a Plausible Antivirulence Target in the Perspective of Emerging Antimicrobial Resistance

**DOI:** 10.3389/fmicb.2017.00070

**Published:** 2017-01-31

**Authors:** Shivangi Sachdeva, Raghuvamsi V. Palur, Karpagam U. Sudhakar, Thenmalarchelvi Rathinavelan

**Affiliations:** Department of Biotechnology, Indian Institute of Technology HyderabadKandi, India

**Keywords:** antibiotic resistance, antivirulence strategy, capsular polysaccharide, *Escherichia coli*, Wzy polymerase dependent pathway, Group 1 CPS, CPS based vaccine

## Abstract

Bacteria evolving resistance against the action of multiple drugs and its ability to disseminate the multidrug resistance trait(s) across various strains of the same bacteria or different bacterial species impose serious threat to public health. Evolution of such multidrug resistance is due to the fact that, most of the antibiotics target bacterial survival mechanisms which exert selective pressure on the bacteria and aids them to escape from the action of antibiotics. Nonetheless, targeting bacterial virulence strategies such as bacterial surface associated polysaccharides biosynthesis and their surface accumulation mechanisms may be an attractive strategy, as they impose less selective pressure on the bacteria. Capsular polysaccharide (CPS) or *K*-antigen that is located on the bacterial surface armors bacteria from host immune response. Thus, unencapsulating bacteria would be a good strategy for drug design, besides CPS itself being a good vaccine target, by interfering with CPS biosynthesis and surface assembly pathway. Gram-negative *Escherichia coli* uses Wzy-polymerase dependent (Groups 1 and 4) and ATP dependent (Groups 1 and 3) pathways for CPS production. Considering *E. coli* as a case in point, this review explains the structure and functional roles of proteins involved in Group 1 Wzy dependent CPS biosynthesis, surface expression and anchorage in relevance to drug and vaccine developments.

## Introduction

Discovery of antibiotics revolutionized modern medicine by protecting millions of people from life-threatening bacterial infections (Ventola, 2015). Though antibiotics underpin important medical advancements in the 20th century, development of resistance against their action to combat bacterial infections is increasingly common in recent years and thus, emerged as one of the greatest threats to human health (Davies and Davies, 2010). Indeed, bacteria develop antimicrobial resistance (AMR) or antibiotic resistance for more than one drug, resulting in multidrug resistance (MDR) that further complicates the issue (Anes et al., 2015). For instance, *Klebsiella pneumoniae*, a ‘superbug’ that is responsible for a range of infections like pneumonia, meningitis, blood stream and surgical infections, is resistant to third-generation cephalosporins and carbapenems (WHO, 2014). WHO’s (2014) report on AMR also states that extensively drug-resistant tuberculosis (XDR-TB) has increased to 9.2% among the reported MDR-TB cases, wherein, drugs like rifampicin, ethambutol, pyrazinamide and isoniazid have become ineffective against the pathogen *Mycobacterium tuberculosis*. MDR is increasingly common in recent years and expensive to treat. It is a great threat to children, elderly and immunocompromised patients such as those who undergo chemotherapy. As per the WHO’s (2014) report, 25000 people in Europe and 23000 in US die annually due to AMR. In fact, a recent report prepared by economist O’Neill (2016) warns that a failure to tackle AMR would claim 10 million lives each year by 2050.

## Mechanisms of Antibiotic Resistance

**FIGURE 1:**
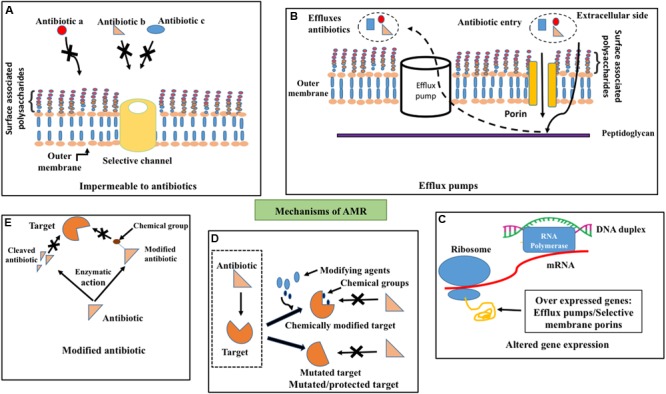
**Antimicrobial resistance mechanisms.** Schematic illustration about **(A)** impermeability for antibiotics (for example, a, b, and c) through outer membrane due to channel (golden colored cylinder) selectivity, **(B)** eﬄux (dotted arrow) of penetrated (solid arrow) antibiotics across the outer membrane (golden colored) by eﬄux pumps (white cylinder) **(C)** altered gene expression such as overexpression of porins that exhibit selectivity for antibiotics and eﬄux pumps **(D)** target site protection through the addition of chemical groups by chemical modifiers (top–right) and mutation in the target site (bottom–right) to prevent the binding of antibiotics to the target (dotted box, left) and **(E)** degradation (left) or chemical modification (right) of antibiotics by enzymatic activity to prevent its binding with the target.

Bacteria exhibit remarkably diverse and complicated mechanisms against the action of antibiotics. Bacteria can evolve antibiotic resistance either through intrinsic or acquired mechanisms (**Figure 1**). Intrinsic resistance is due to the intrinsic functional or structural characteristics of the bacterium. For example, due to the difference in the membrane structure of Gram-positive and Gram-negative bacteria, lipopeptide daptomycine is good for treating the former, whereas it is unsuitable to treat the latter due to inefficiency of daptomycine to get inserted into the outer membrane (Randall et al., 2013) (**Figure 1A**). Though the antibiotic can enter the outer membrane in some cases, the bacteria can actively pump it out by eﬄux pumps (Webber and Piddock, 2003) before it reaches the target (**Figure 1B**). This mechanism contributes to intrinsic resistance mainly in Gram-negative bacteria due to the presence of membrane spanning nanomachineries such as resistance-nodulation-division (RND) eﬄux pumps (Venter et al., 2015). Due to these reasons many drugs that are effective in Gram-positive bacteria are ineffective in Gram-negative bacteria.

## Acquired Resistance

Bacteria acquire resistance to antibiotics through chromosomal gene mutations known as vertical evolution that can subsequently be transmitted to other bacterial strains through horizontal gene transfer (HGT) (Poole, 2011; Radhouani et al., 2014; von Wintersdorff et al., 2016). Three main actions come under acquired resistance (**Figures 1C–E**): (1) preventing the target access by reducing the intracellular accumulation of antibiotics through poor penetration or increased eﬄux (Zgurskaya et al., 2015), (2) alternations in antibiotic target by genetic mutation or post-translational modification and (3) inactivation of antibiotics through chemical modification (Blair et al., 2015).

One such example for aforementioned antibiotic refractory mechanism is resistance to clinically effective antibiotics by overexpression of eﬄux pumps (Anes et al., 2015; Alcalde-Rico et al., 2016) (**Figure 1C**) as found in patients infected with Enterobacteriaceae family of Gram-negative bacteria (Everett et al., 1996)*, Staphylococcus aureus* (Kosmidis et al., 2012) etc. (Pumbwe and Piddock, 2000). Permeability of the antibiotics (**Figure 1A**) can also be reduced through the down-regulation of porins as in Enterobacteriaceae (Lavigne et al., 2013), which results in resistance to antibiotics like carbapenems (Baroud et al., 2013). Such antibiotic resistance gene(s) can be mobilized onto plasmids and transferred between bacteria.

Target site protection (**Figure 1D**) is yet another mechanism that bacteria adopt to escape from the antibiotic activity by changing their target structure, without interfering with their function, which consequently prevents the specific binding of antibiotic to the target. Indeed, even a point mutation in the gene can confer antibiotic resistance that can subsequently proliferate within the strain. Fluoroquinolone resistance in Enteroaggregative *Escherichia coli* (EAEC) (Kim et al., 2012) is also due to single or multiple mutations in quinolone resistance determining regions (Taneja et al., 2015). Target change can also be achieved through the acquisition of a homologues gene that is similar to the target gene. This resistance gene can also be disseminated across various strains and species through horizontal gene transfer through DNA uptake from the environment. Addition of chemical groups to the target site (**Figure 1D**) also offers resistance to antibiotics. For instance, chloramphenicol-florfenicol resistant (crf) methyltransferase (Shen et al., 2013) in *E. coli*.

Bacteria can also escape the antibiotics by destroying the antibiotics or modifying the structure by transfer of chemical groups to the antibiotics (**Figure 1E**). Enzymes such as β-lactamase (Abraham and Chain, 1988), carbapenemase (Tzouvelekis et al., 2012), imipenemase and New Delhi metallo-β-lactamase (Nordmann et al., 2011; Johnson and Woodford, 2013) carry out hydrolysis of a large spectrum of antibiotics (Livermore, 2008) in Gram-negative bacteria.

Besides antibiotic resistance, bacteria can also exhibit resistance against biocides such as antiseptics, disinfectants and sanitizers due to their widespread use, by applying similar mechanisms as against antibiotics (Russell, 1999; Poole, 2002; Morita et al., 2014). Possible links between biocide resistance and antibiotic resistance are a major concern as it could exacerbate situation of increased resistance in clinically relevant organisms (Fraise, 2002).

## Evolution of Antibiotic Resistance and its Dissemination

Antimicrobial resistance is a natural phenomenon that bacteria exhibit against natural antibiotics for their survival (Sheldon, 2005). Antibiotic resistance gained attention during the mid of 20th century with the development of resistance against the first antibiotic penicillin. Many drugs have become ineffective soon after their arrival on the market due to their overuse and misuse, which have led to the global health crisis (Unemo and Nicholas, 2012).

Clinical, agricultural and environmental factors (Cattoir et al., 2007) are the three major sources that facilitate the development of antibiotic resistance. Intensive use of a broad range of antibiotics in human, agricultural and veterinary medicine without precise diagnostics exerts selective pressure on the bacteria, which can give rise to a gene that confers resistance to an antibiotic. One such example is emergence of mutations in porin genes in *E. coli* due to carbapenem exposure (Tangden et al., 2013).

Other sources that aid in the emergence of antibiotic resistance are laboratories carrying out genetic manipulation, pharmaceutical industries, wastewater habitats, poultry wastes and livestock wastes. Pharmaceutical industries, wherein, microorganisms are involved in the production of antibiotics, expose the local microbiota to the antibiotic and motivate AMR gene selection. Residual antibiotics present in poultry and livestock wastes, sewage treatment plants exert pressure for the selection of AMR genes (Fletcher, 2015). Though wastewater treatment plants reduce the AMR bacteria, it may lead to MDR (Cantas et al., 2013).

Evolved AMR gene(s) can subsequently be disseminated to human pathogens through food (Founou et al., 2016), environment (Radhouani et al., 2014) and direct or indirect contact with livestock industry (Singer et al., 2016) through horizontal gene transfer. One such evidence is transmission of resistance genes from aquatic environment: similarity between AMR genes in fish (*Aeromonas*) and human (*E. coli*) pathogens (Sørum, 2006). Similarly, OXA-48-type carbapenem-hydrolyzing β-lactamase genes leading to resistance among Enterobacterial species have been found to emerge from the chromosome of *Shewanella* species (Poirel et al., 2012). Among the mechanisms of HGT, conjugation is the most prominent route, wherein, DNA is transferred in a multi-step process demanding cell to cell contact through pili or adhesins along with other mechanisms like transformation and transduction (von Wintersdorff et al., 2016). For instance, ampicillin resistance in members of the Enterobacteriaceae family and tetracycline resistance in enterococci are observed due to plasmid or transposon mediated conjugation (Rice, 1998). Transformation is another mechanism for HGT, in which, specific bacterium is capable of up taking extracellular DNA (that posses antibiotic resistance) from natural reservoir and develops resistance. For example, *in vitro* experiments have revealed that *gyrA* and *parC* genes responsible for fluoroquinolone resistance are transformed between *Streptococcus pneumoniae* (Ferrandiz et al., 2000) and several *Streptococci viridans* (Gonzalez et al., 1998). It has been speculated that transduction may be playing an important role in generating novel methicillin-resistant *Staphylococcus aureus* (MRSA) strains (Barlow, 2009).

Cross-border and cross-continental human mobility (Arya and Agarwal, 2011; Cheng et al., 2012) is also one of the causes for the spreading of bacterial AMR gene as seen in *K. pneumoniae* ‘resistance gene movement’ (McKenna, 2013). *K. pneumoniae* carbapenemase (KPC)-producing bacteria that were initially found in North Carolina (2000) soon spread to New York in 2003. By 2005, the resistance strain was found in Israel, from where, it traveled to Italy, Colombia, United Kingdom and Sweden.

## Bacterial Virulence Mechanisms and Anti Virulence Strategies

As the use and misuse of antibiotics have lead to the selection of antibiotic resistant organisms, it mandates the development of alternative strategies to treat bacterial infections (Cantas et al., 2013). In general, bacteria evolve resistance against a drug because antibiotics are designed to either kill the bacteria or prevent their growth (Kohanski et al., 2010) by disrupting the biosynthesis and assembly of an essential cellular pathway. This exerts substantial stress on the bacterium to evolve AMR gene. Considering these, one can think of alternative strategies that can reduce the selective pressure on the bacteria by interfering with the mechanisms that are not essential for their survival such as bacterial virulence and intercellular communications.

**FIGURE 2:**
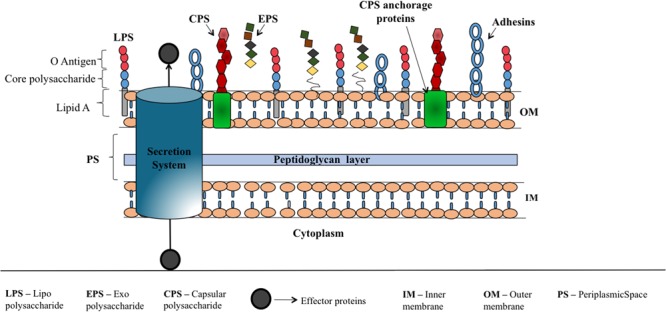
**Schematic illustration corresponding to various virulence factors of Gram-negative bacteria.** Bacterial surface associated polysaccharides that are covalently or non-covalently bound to outer membrane proteins (CPS), linked to lipid A (LPS) and loosely attached to the surface (EPS) which help the bacteria to escape from host immune response, aid in bacterial colonization, offer resistance for penetration of antibiotics etc. are shown along with adhesins that facilitate the attachment of bacteria onto the host cell. Secretion systems (I–VII) (Rasko and Sperandio, 2010; Depluverez et al., 2016) that deliver proteins and DNA to extracellular region or into the target cells is represented by a cylinder.

Soon after the bacteria detects the host cell by recognizing host signals in the environment, they initiate the self-defense mechanism to activate the virulence traits to attach and invade the host, evade host immune response and infect the host. Major bacterial virulence factors (**Figure 2**) are: toxins, adhesins (Rasko and Sperandio, 2010), capsular polysaccharides (CPS), lipopolysaccharide (LPS) and exopolysaccharide (EPS) that aid the bacteria to attach onto the host cell, escape from host immune response, cloak sub capsular epitopes on the bacterial surface and colonize (Wu et al., 2008). These components are being exported to the bacterial surface or host cell using sophisticated proteinaceous nanomachines like type I to type VII secretion systems (Abdallah et al., 2007; Rasko and Sperandio, 2010; Filloux, 2011) and CPS, LPS and EPS exportation systems. Thus, interfering with the mechanisms that bacteria use for sensing the host or preventing the expression or action of virulence factors could control bacterial virulence. Consequently, the host can be able to combat the infection through its immune response.

Aforementioned virulence factors utilize diverse supramolecular nanomachines for their surface expression and attachment. This mandates knowledge about the structure of nanomachinery components and intercomponent interaction mechanisms to establish the molecular architecture of nanomachines and the concomitant virulence factor exportation mechanisms.

In this review, we focus on the structure and assembly of *E. coli* CPS or *K*-antigen exportation machinery that facilitates the surface expression of CPS. As CPS is crucial for bacterial pathogenicity, it can be a good vaccine/drug target.

## Escherichia coli

*Escherichia coli* is a Gram-negative bacteria that belongs to Enterobacteriaceae family and is a common cause for urinary tract infections (including infections of the kidney), bloodstream infection, diarrhea (Croxen et al., 2013) foodborne infections, intra-abdominal infections such as peritonitis, skin and soft tissue infections, and neonatal meningitis. Periodic outbreaks of *E. coli* strains such as O121, O26, O104, O145, and O157 take place, including the outbreak of *E. coli* O157 that happened in 2016 through alfalfa onion sprout products in the US^1^^,^^2^. Due to its genetic flexibility and adaptability to constantly changing environments, *E. coli* easily acquires a great number of AMR mechanisms, thus, treating the infections become difficult (Szmolka and Nagy, 2013). For instance, Center for Disease Control and Prevention (CDC) has listed O157:H7 strain of *E. coli* as a potential bioterrorism agent. Survey among 194 WHO member states in 2014 clearly indicates that *E. coli* has evolved resistance against third-generation cephalosporins and fluoroquinolones, the two major antibacterial drugs that are currently used to treat *E. coli* infections (WHO, 2014). Reports on ‘emergence of carbapenem resistance’ in *E. coli* (Hong et al., 2005) are quite alarming, as it is the antibiotic of ‘last resort’ (Papp-Wallace et al., 2011).

## Cell Surface Associated Glycoconjugates in *E. coli*

Like many other Gram-negative bacteria, cell surface glycoconjugates encapsulate *E. coli* and play crucial role in interaction with the environment. Three major groups of secreted polysaccharides are LPS (Putker et al., 2015), CPS or *K*-antigen (Whitfield, 2006) and EPS (Whitfield et al., 2015). LPS consists of lipid A, core polysaccharides and *O*-antigen polymer, wherein lipid A moiety facilitates the covalent insertion of LPS into the outer leaflet of outer membrane (**Figure 2**). CPSs are high-molecular weight polysaccharides that are covalently/non-covalently attached to cell. On the other hand, EPS is loosely in association with the cell surface, and are usually secreted to the extracellular environment to facilitate biofilm formation (Yuan et al., 2013). Among these three surface associated polysaccharides, *O* and *K*-antigens are serotype-specific. In *E. coli*, the combination of ∼170 *O*-antigens and ∼80 *K*-antigens along with *H*-antigens gives rise to 50,000–100,000 or more different serotypes (Ørskov and Ørskov, 1992; Whitfield, 2006), among which some are pathogenic.

## Capsular Polysaccharides

**FIGURE 3:**
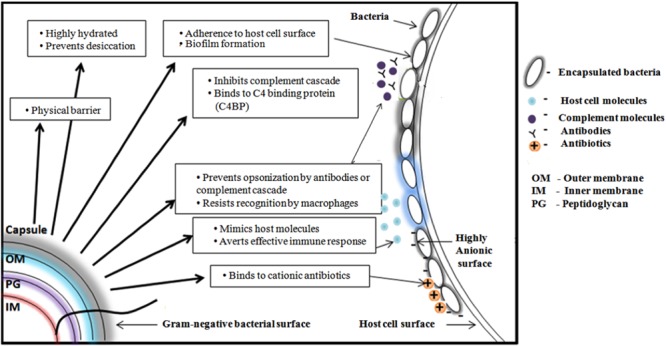
**Schematic representation of various functions of Gram-negative bacterial capsular polysaccharide.** From clockwise direction: Offering physical barrier to antibiotics, preventing bacterial cell desiccation due to hydrated nature, facilitating the bacteria to adhere onto host cell surface, helping the bacteria to escape from host immune response by inhibiting complement cascade, preventing antibody opsonization, precluding the recognition by macrophage, mimicking host molecules and inhibiting the penetration of cationic antibiotics by binding with it.

*K*-antigens are one of the major virulence determinants of *E. coli* that help the bacteria to escape from the bacterial host immune response (Whitfield, 2006). The degree of virulence depends on the size and composition of the capsule (Horwitz and Silverstein, 1980). CPS plays a role in defending bacteria from phagocytosis that is a part of host’s innate immune response. It prevents activation of the phagocytic process by decreasing the binding of opsonins, and by masking ligands for phagocytic cell attachment. For example, the *E. coli* K1 antigen binds to C4b-binding protein (C4BP) – a major inhibitor of the classical complement pathway (Maruvada et al., 2008). Capsules can mask the underlying antigenic structures of the bacteria, thereby making them less susceptible to the host’s immune system. CPS could be structurally similar to host cell polysaccharides and thereby a host could tolerate the bacteria owing to poor immunogenicity. For example, K1 CPS is structurally identical to neural cell adhesion molecule (N-CAM) (Roberts, 1996). It further aids the bacteria to adhere to the host and also in biofilm formation. Cationic antimicrobial peptides have microbicidal properties against many pathogens (Fleitas and Franco, 2016). These peptides disrupt the bacterial membranes and lead to lysis of the cell. CPS, being anionic binds these peptides and prevents the cell lysis (Band and Weiss, 2015). **Figure 3** illustrates all the above-discussed mechanisms.

Capsular polysaccharide also facilitates AMR in Gram-negative bacteria by offering barrier for antibiotics to diffuse into the host cell. A role of CPS in offering resistance to antibacterial peptide and proteins in Gram-negative bacteria is well established (Campos et al., 2004; Llobet et al., 2008). For instance, increased exposure to antibiotic peptides increased the production of CPS by upregulating the expression of the associated genes. Sublethal use of kanamycin and streptomycin in *E. coli* have been demonstrated to increase CPS production and thickness that eventually reduced the penetration of antibiotics into the cell (Lu et al., 2008). Several successful attempts have been made to inhibit the biogenesis of CPS using anticapsule small molecule inhibitors in *E. coli* (Goller and Seed, 2010). CPS based vaccines against *E. coli* infections are also underway (Brumbaugh and Mobley, 2012; Hutter and Lepenies, 2015; Mobley and Alteri, 2015).

*Escherichia coli K*-antigens can be classified into four groups on basis of genetic and biochemical criteria, namely, Group 1, Group 2, Group 3, and Group 4. While Groups 1 and 4 utilize Wzy-dependent pathway, Groups 2 and 3 use ATP-dependent pathway for surface exportation and anchorage of *K*-antigen. We aim to provide insights about the structure, function and assembly of proteins involved in Wzy-dependent Group 1 *K*-antigen exportation pathway. As the CPS exportation machinery spans the inner and outer bacterial membranes, it can be easily accessible for the drug molecules and thus, can be a good target. Additionally, disrupting the surface anchorage of CPS is an attractive strategy. Thus, detailed molecular insights about Group 1 *K*-antigen exportation machinery would ease the anticapsule drug design. Nineteen *K*-antigens, namely, K26, K27, K28, K29, K30, K31, K32, K33, K34, K35, K36, K37, K39, K41, K42, K43, K55, K102, and K103 come under Group 1 classification and are negatively charged due to the presence of uronic acid and/or pyruvate (Kunduru et al., 2016).

## Overview of the Wzy-Dependent Pathway

**FIGURE 4:**
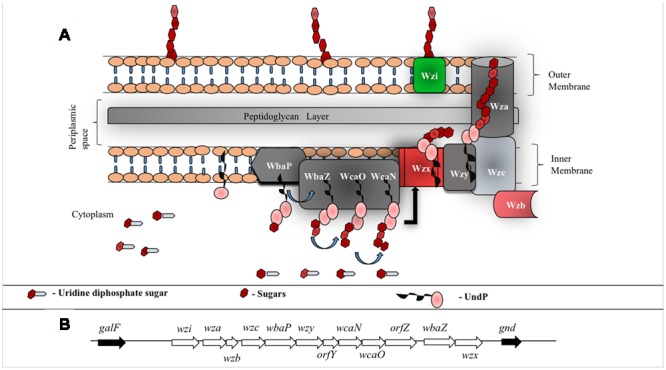
**(A)** Schematic representation of group 1 CPS biosynthesis and surface exportation machinery. WbaP (initiating transferase), WbaZ (glycosyltransferases (GT)), WcaN (GT) and WcaO (GT) located in the inner membrane and aids in biosynthesis of CPS repeating unit. Wzx, a flippase, is involved in flipping the repeat unit from cytoplasmic region to periplasmic space and Wzy, a copolymerase, participates in the polymerization of oligosaccharide repeating unit. Inner membrane proteins, Wzb (phosphatase) and Wzc (tyrosine autokinase) work synergistically with outer membrane protein Wza (translocon) to export CPS to the bacterial surface. Wzi (lectin and porin) anchors CPS onto the bacterial outer membrane. **(B)** *E. coli* group 1 K30 CPS locus located in between *galF* and *gnd* on E69 chromosome (Rahn et al., 1999). *wbaP, wbaZ, wcaN* and *wcaO* code for transferases that are involved in the biosynthesis of CPS repeating unit, *wzx* and *wzy* code for proteins involved in polymerization and *wza, wzb* and *wzc* code for proteins involved in translocation of CPS across membranes. *wzi* (formerly called *orfX)* codes for protein that facilitates anchorage of the polysaccharide onto the outer membrane. Functions of *orfY* and *orfZ* remain unclear.

Surface expression of *K*-antigen is a multistep process (**Figure 4A**) that commences in the cytoplasm by attaching the nucleotide diphospho sugar precursor [commonly, uridine diphosphate (UDP) attached with the corresponding hexopyranose monosaccharide of the *K*-antigen] to the undecaprenyl phosphate (Und-P) carrier lipid at the cytoplasmic face of the inner membrane. Subsequently, the other monosaccharaides of *K*-antigen repeating unit are sequentially glycosylated to form hexopyranose-PP-UndP with the help of glycosyltransferases (WbaZ, WcaO and WcaN) (Whitfield and Paiment, 2003). Lipid linked oligosaccharide is then transported across the inner membrane and the repeating unit is polymerized at the periplasmic region with the help of an integral membrane protein Wzy, hence, acquiring the name Wzy-dependent pathway (Whitfield, 2010), synonymously, Wzx/Wzy pathway (Whitney and Howell, 2013) (**Figure 4A**). Strikingly, EPS also utilizes Wzy dependent pathway for its surface expression (Whitney and Howell, 2013). Thus, understanding the Wzy-dependent pathway may shed light not only on CPS surface expression, but also, on EPS surface expression. As CPS also undergoes chemical modifications akin to EPS (that eventually increases bacterial pathogenicity), detailed insights about the Wzy pathway is warranted to understand the mechanism behind the surface expression of chemically modified CPS and its role in bacterial virulence (Whitfield et al., 2015). In this context, this review explains *E. coli K*-antigen surface expression by considering K30 as a case in point.

In total, there are 12 proteins involved in K30 antigen repeat biogenesis, polymerization, transportation and its surface anchorage (**Figure 4**). The genes responsible for these proteins are clustered together in a 16 kb cluster called *cps* (**Figure 4B**). While the genes upstream in the cluster code for high level polymerization, export and assembly of CPS, the genes downstream code for the proteins involved in CPS repeat unit synthesis and polymerization (Rahn and Whitfield, 2003). This process happens in a sequential manner through proteinaceous supramolecular nanomachinery (Drummelsmith and Whitfield, 1999; Rahn et al., 2003). Below are the known structural and functional insights about all the proteins involved in Wzy-dependent pathway.

## WbaP: a Transferase

**FIGURE 5:**
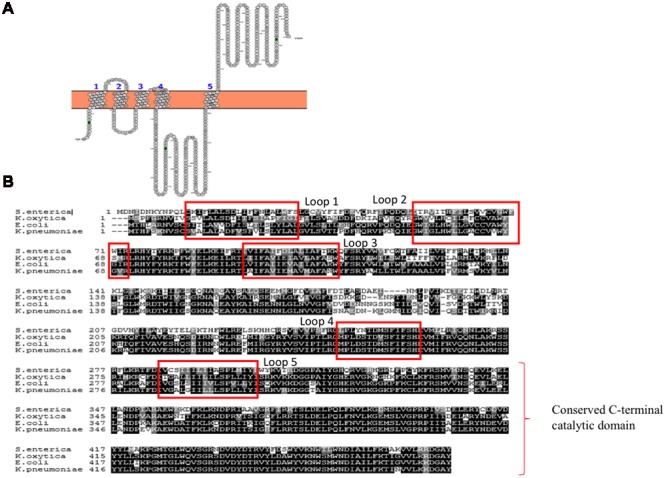
**(A)** Topology map of WbaP of *E. coli* K30 serotype (ACCESSION: AAD21565). It is predicted to have five transmembrane helices in accordance to prediction made for WbaP of *Salmonella enterica* (Saldias et al., 2008). Note that topology maps are predicted and generated using Protter server (Omasits et al., 2014). **(B)** Multiple sequence alignment showing highly conserved nature of WbaP among *S. enterica, E. coli, K. pneumoniae*, and *K. oxytoca.*

WbaP is an integral membrane protein that belongs to polyisoprenyl-phosphate hexose-1-phosphate transferases (PHPTs) family of enzymes that initiates the assembly of *K*-antigen components at the cytoplasmic face of the inner membrane. WbaP is involved in initiation of *O*-antigen synthesis in *Salmonella enterica* and CPS biosynthesis in *E. coli* K30 (Patel et al., 2012b). It catalyzes the transfer of galactose-1-phosphate sugar (Gal-1-P) from uridine diphospho galactose (UDP-Gal) by establishing phosphoanhydride bond formation with undecaprenyl-phosphate (Und-P) lipid carrier with concomitant release of uridine monophosphate (UMP) (Patel et al., 2010, 2012a). Based on the topology and functional studies of *S. enterica* WbaP corresponding to Wzy-dependent *O*-antigen biosynthesis and surface expression (Patel et al., 2010), one can envisage that *E. coli* Group 1 WbaP can also have five transmembrane segments (**Figure 5A**) based on the sequence alignment (**Figure 5B**). As in *S. enterica*, while the N-terminal domain help in insertion of the protein into the inner membrane, the C-terminal domain regulates the Gal-1-P transferase activity and specificity for Und-P (Saldias et al., 2008; Patel et al., 2012a). As WbaP has no eukaryotic homolog (Saldias et al., 2008), it can be a good drug candidate.

## Wzx: a Flippase

The Und-P attached *K*-antigen repeating unit is transported across the inner membrane with the help of an integral inner membrane protein Wzx, a flippase (Whitfield, 2006). It belongs to the family of polysaccharide-specific transport (PST) proteins and is designated as PST-1 due to its involvement in translocating the *K*-antigen repeating unit from the cytoplasmic leaflet to periplasmic leaflet. Hitherto, there is no experimentally derived 3-dimensional structural information available for *E. coli* Group 1 *K*-antigen Wzx. However, an earlier study about *O*-antigen specific Wzx (Marolda et al., 2010) has highlighted significant features about the topology and functionally critical amino acids of Wzx involved in *O*-antigen transport in *E. coli* O157. The study has also identified the importance of D85, D326, R298, and K419 by mutating to alanine or serine that resulted in the expression of Wzx that is unable to support *O*-antigen production (Marolda et al., 2010). Further, this study has proposed that charge and spatial position of these critical residues are important to let the Und-PP-linked oligosaccharide to translocate.

Wzx falls under 1 of 4 families in multidrug/oligosaccharidyl-lipid/polysaccharide (MOP) superfamily (Hvorup et al., 2003). Thus, a very recent study (Islam et al., 2013) has modeled the structure of Wzx involved in LPS surface exportation in *Pseudomonas aeruginosa* using the structure of NorM protein of *Vibrio cholera* O1 El Tor, a multidrug and toxin extrusion (MATE) family of inner membrane eﬄux protein, as a template (Islam et al., 2010, 2012). The authors have used the previously mentioned similarity between MATE family and PST family proteins to propose a model for Wzx flippase activity in *P. aeruginosa* and suggested antiport mechanism for UndPP-linked oligosaccharide flipping (Islam and Lam, 2013).

**FIGURE 6:**
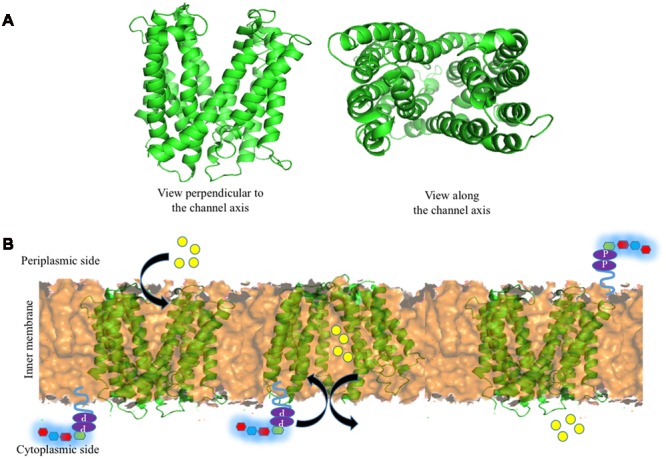
**Structure of Wzx and the associated Und-P (colored purple) attached oligosaccharide flipping across the inner membrane (golden colored surface). (A)** Cartoon representation of I-TASSER (Yang et al., 2015) predicted structure of *E. coli* K30 Wzx by using NorM protein from *V. cholera* (PDB 3MKU) as a template. **(B)** Schematic representation of oligosaccharide repeat ‘antiport’ flipping mechanism by Wzx as proposed by Islam and Lam (2013). H^+^/Na^+^ ions (yellow spheres) located in (left) the periplasmic side binds to Wzx (middle), and subsequently opens up the cytoplasmic side of Wzy and moves toward cytoplasmic side by exchanging Und-P attached oligosaccharide repeating unit to the periplasmic side (right).

Encouraged by the above, Group 1 K30 Wzx is modeled here (**Figure 6A**) using fold recognition based server I-TASSER (Yang et al., 2015). The model is quite similar to the *O*-antigen Wzx of *P. aeruginosa* with 12 transmembrane helices, despite the poor sequence identity of 13%. Thus, as proposed by Islam and Lam (2013), the antiport mechanism may also be applicable here, specifically in the context of negatively charged Group 1 polysaccharide (**Figure 6B**). Cation (H^+^/Na^+^) binding to periplasmic face of the Wzx brings conformational changes, that subsequently facilities the opening of cytoplasmic face, and the binding of UndPP-linked oligosaccharide to Wzx. Upon UndPP-linked oligosaccharide binding, Wzx reverts back to periplasmic open state conformation and releases UndPP-linked oligosaccharide to inner membrane leaflet. This is concomitant with the release of Wzx bound cations to cytoplasm.

## Wzy: a Polymerase

**FIGURE 7:**
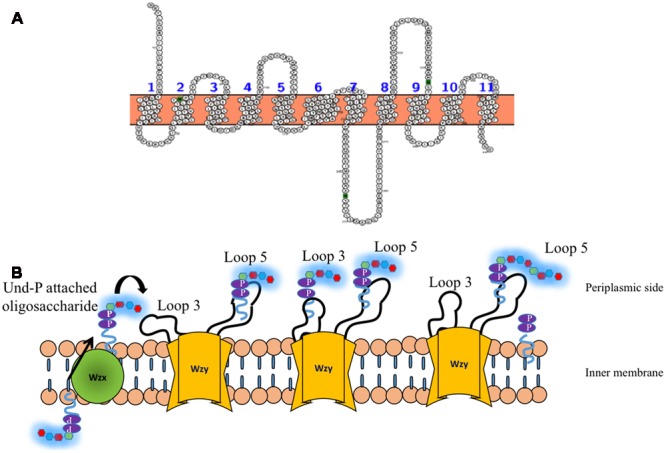
**(A)** Topology map of Wzy *E. coli* K30 serotype (ACCESSION: AAD21566) is predicted to have eleven transmembrane helices. **(B)** ‘Catch and release’ mechanism involved in the oligosaccharide polymerization by Wzy (golden colored) (Islam et al., 2011). (Left) Und-P attached oligosaccharide that is flipped to periplasmic side by Wzx (**Figure 6**) is fetched by loop 3 of Wzy (left, solid arrow) and undergoes conformational changes so as to bring the oligosaccharide repeating unit close to loop 5 (Middle), wherein, the nascent CPS is being held. Consequently, the oligosaccharide repeating unit is transferred from loop 3 and attached to the nascent CPS by leaving Und-PP (Right). The above reaction takes places in a cyclic manner through distributing mode.

Wzy is the polymerase that is present in the inner membrane (Whitfield, 2010) and has been suggested to have 9–12 transmembrane segments depending on the serotype along with a large periplasmic loop (**Figure 7A**) (Daniels et al., 1998; Islam et al., 2010). It has been shown that in the case of *E. coli* K30 serotype, knockout of Wzy lacks CPS, implicating its importance in CPS surface expression (Drummelsmith and Whitfield, 1999). Wzy is required for polymerization of undecaprenyl diphosphate-linked *K*-antigen sugar moiety in the periplasmic region. It has been proposed that Wzy polymerizes the repeating oligosaccharide that has been translocated by Wzx in a blockwise manner (Whitfield, 2006). Subsequently, it releases the *K*-antigen oligosaccharide sugar moiety from its lipid carrier to the nascent polymer (Schild et al., 2005). This coupled functionality of Wzx translocation and Wzy polymerization in the context of surface associated polysaccharides has lead to name “Wzx/Wzy pathway” (Schmid and Sieber, 2015) or “Wzx/Wzy secretion system” (Whitney and Howell, 2013). A recent study on O86 antigen polymerization in *E. coli* Wzy dependent pathway proposes that Wzy adopts a distributive mode of repeat oligosaccharide polymerization, wherein, after each round of repeat polymerization the enzyme dissociates from the nascent CPS and randomly binds to the repeat oligosaccharide to carry out the reaction in a cyclic manner (Zhao et al., 2014). Another study carried out on *P. aeruginosa* PAO1 suggests a “catch and release” model (**Figure 7B**). According to this model, the periplasmic loop 3 captures the repeating oligosaccharide and periplasmic loop 5 performs the polymerization and substrate release activities (Islam et al., 2011, 2013).

## Wzc: an Inner Membrane Tyrosine Autokinase

**FIGURE 8:**
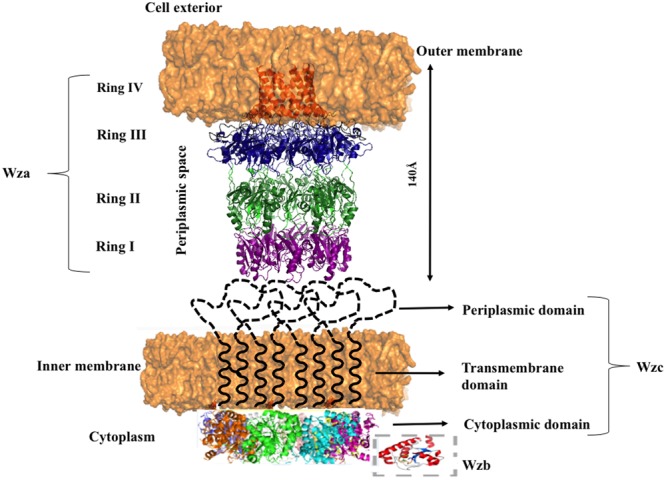
**Diagram illustrating the interacting mode of Wza, Wzc, and Wzb that are involved in CPS surface exportation.** While Wza (PDB ID: 2J58) spans the periplasmic and outer membrane regions, Wzc spans periplasmic, inner membrane and cytoplasmic regions. Crystal structure of Wzc cytoplasmic domain (PDB ID: 3LA6) is represented in cartoon, whereas, its periplasmic and inner membrane regions are represented by black colored dotted loops and solid helixes respectively. In line with the ocatmeric form of cytoplasmic Wzc, periplasmic and inner membrane regions are also given as octamers. Wzb located in cytoplasmic region is shown in dotted box. It is to be noted that membrane is shown in orange surface representation. Note: Wza- Wzc interaction is based on model proposed by Collins et al. (2006).

Nascent *K*-antigen is then translocated onto the bacterial surface by synergetic action of Wza, Wzc, and Wzb (Cress et al., 2014), (**Figures 4A** and **8**) whose length and amount are controlled by Wzc and Wzb (Morona et al., 2000; Obadia et al., 2007). Wzc, is a bacterial tyrosine (BY) autokinase that also functions as a copolymerase by regulating the length of the polymer together with Wzb, its cognate phosphatase. *wzc* gene codes for a copolymerase that belongs to polysaccharide copolymerase 2a (PCP-2a) subfamily which is required for the assembly of Group 1 capsule (Cuthbertson et al., 2009). Though Wzy is capable of polymerizing the sugar moieties, Wzc and Wzb are shown to be essential for longer *K*-antigen structure (Collins et al., 2006).

**FIGURE 9:**
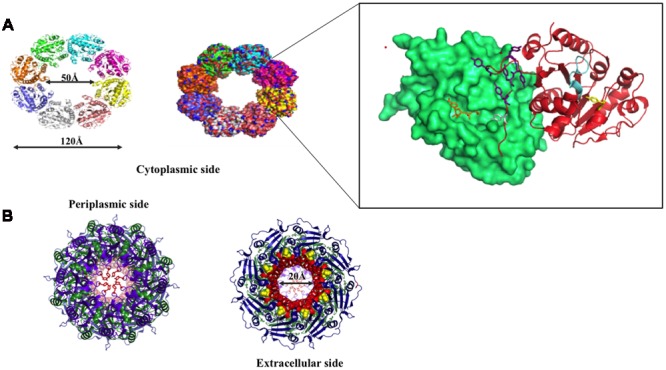
**Wzc and Wza structures. (A)** Top view of Wzc cytoplasmic domain is shown in cartoon (right) and surface (left) representations. Two interacting Wzc monomers are magnified (boxed) to show the Walker A motif (ATP binding site; represented cyan), Y569- the first autophosphorylated tyrosine (represented in yellow stick) and C-terminal Y-cluster (blue colored stick) along with the first transphosphorylated Y715 that is inserted into the adjacent monomer. ADP (orange colored stick) bound to its Walker A motif site is also shown. **(B)** Extracellular (right) and periplasmic (left) views of Wza. Trp350 on extracellular side (right) and Tyr110 at periplasmic side (left) are indicated by yellow colored spheres and orange colored sticks respectively. Note that Tyr110 seals the periplasmic entry.

Wzc is an integral membrane protein consisting of two transmembrane helices (32–52 and 426–445 residues) flanking a large periplasmic domain (53–425 residues) and a cytoplasmic C-terminal region (446–720 residues) (Doublet et al., 2002) containing an extended tyrosine-rich tail (seven tyrosine residues within last 17 amino acids). Cytoplasmic domain (**Figure 9A**) has Walker A and B motifs which are involved in ATP binding and hydrolysis (Wugeditsch et al., 2001; Cuthbertson et al., 2009). The domain also has a phosphate binding loop (P-loop), a general characteristic of nucleotide binding P-loop proteins.

Wzc autophosphorylates the C-terminal tyrosine cluster (Y-cluster) to regulate the polymerization of the nascent *K*-antigen. This happens in association with oligomerization of Wzc. The process is initiated with autophosphorylation of Y569 and followed by transphosphorylation of Y-clusters in neighboring monomers (Wugeditsch et al., 2001; Standish and Morona, 2014). However, the precise mechanism about how autophosphorylation of Wzc in cytoplasmic C-terminal Y-cluster controls the length of *K*-antigen in the periplasmic region is poorly understood. Wzc knockout or compromise on its phosphorylation such as deletion of Y-cluster is shown to halt the polymerization and surface expression of *K*-antigens (Paiment et al., 2002).

In the crystal structure of cytoplasmic tyrosine autokinase domain of *E. coli* K12, Wzc exists in ring-shaped octameric form with Y-cluster in non-phosphorylated state (Bechet et al., 2010). In **Figure 9A**, Wzc is complexed with ADP, such that the tyrosine rich region of one monomer is inserted onto the active site of another. This has led to the assumption that in the non-interactive state of the Wzc cytoplasmic domains, Y-cluster is in phosphorylated state that could be easily dephosphorylated by Wzb to promote interaction between Wzc cytoplasmic domains (Wugeditsch et al., 2001; Standish and Morona, 2014). Nonetheless, divergent evidence exists about the relationship between phosphorylation (non-interactive)/dephosphorylation (interactive) of Wzc and its copolymerase activity (Wugeditsch et al., 2001; Standish and Morona, 2014). While some support that phosphorylated Wzc promotes polymerization, others suggest the reverse. In any case, cycling between the two states is essential to regulate the *K*-antigen biosynthesis.

Transmission electron microscope (TEM) map of Wzc-Wza complex (Collins et al., 2006, 2007) shows that Wzc directly interacts with octameric Wza in oligomeric form at the periplasmic region (Wugeditsch et al., 2001). In Wzc-Wza complex TEM map, Wzc resembles an extracted molar tooth where the crown is formed by periplasmic region and cytoplasmic tyrosine autokinase domain makes up the roots (Collins et al., 2006). However, more detailed structural investigation is warranted due to the difference in the level of oligomerization of Wzc: octameric crystal structure (Bechet et al., 2010) versus tetrameric TEM structure (Collins et al., 2006, 2007). The level of phosphorylation of the C terminal tyrosine residues (seven residues) may determine the oligomerization state of Wzc.

## Wzb: Phosphotyrosine Phosphatase

Degree of polymerization of nascent *K*-antigen is controlled by the combined action of Wzc and Wzb, a cognate low molecular weight phosphotyrosine phosphatase (LMW PTP) (Standish and Morona, 2014). Located in cytosol, Wzb dephosphorylates the tyrosine residues present in the Wzc Y-cluster. However, detailed mechanism behind this process is not known, due to the lack of structural information about Wzc-Wzb complex. A model suggests that for continued polymerization of the sugar repeating units, phosphorylation and dephosphorylation of C-terminal tyrosine residues of Wzc by Wzb are required (Whitfield, 2006). Indeed, mutation in Wzb resulted in an acapsular phenotype (Wugeditsch et al., 2001), implicating its importance in capsule surface expression.

**FIGURE 10:**
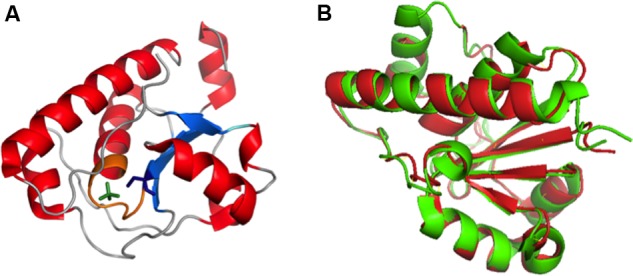
**(A)** Crystal structure of Wzb of *E. coli* K30 serotype bound with PO4 (PDB ID: 2WJA). Nucleotide binding P loop is colored in orange. PO4 and Cys13 are represented in green and blue colored sticks respectively. **(B)** Superimposed structures of Wzb of K30 (crystal structure, PDB ID: 2WJA, colored red) and K12 (Structure derived from NOE based distance restraints and RDC based orientational restraints, PDB ID: 2FEK, colored green) serotypes exhibit high structural similarity with root mean square deviation of 0.8 Å (Hagelueken et al., 2009a). Thus, the phosphatase mechanism proposed for K12 Wzb can also be applicable to K30 Wzb.

Crystal structure (PDB:2WJA) of K30 *E. coli* Wzb coincides well with typical LMW PTP of eukaryotes with 4 β–strands (β 1–4) that consists of residues Ser8-Cys13, Lys34–Gly40, Leu80–Val85 and Thr104–Leu106 respectively and five α-helices (Hagelueken et al., 2009a) (α 1–5) that encompasses residues Cys18–Arg29, Asp50–Arg55, Thr73–Arg78, Glu87–Gly103 and Ser124–Lys145 respectively. The tertiary structure has parallel β strands surrounded by α1, α2, α5 on one side and α3, α4 on the other side with three loops connecting β2-α2, α2-α3 and β4-α5. The structure also reveals the presence of C(X)_5_RS motif (P-loop), a hallmark of cysteine-dependent PTP superfamily. The crystal structure of phosphate bound *E. coli* Wzb (**Figure 10A**) clearly shows that cysteine 13 located in the active site cavity is the catalytic nucleophile that attacks the phosphate group of tyrosines in Wzc and dephosphorylates it. Catalytic mechanism proposed involves conserved positively charged Arg 19 which binds to incoming phosphate, followed by the displacement of tyrosine by the Cys 13 thiol and protonation of tyrosine is done by Asp119 or Cys18 (Hagelueken et al., 2009a). The final step involves hydrolysis of thiophosphate ester by a water molecule, activated by Asp119, leading to the generation of free phosphate and the regeneration of the thiol in Cys 13.

K30 *E. coli* Wzb structure (PDB:2WJA) exhibits significant structural similarity with K12 *E. coli* Wzb (PDB: 2FEK) (Lescop et al., 2006) with 0.8Å root mean square deviation (**Figure 10B**). The study also reveals through NMR based phosphate titration experiments that phosphate binding Wzb leads to structural rearrangement in Cys13 and other coordinating residues (Cys9–Asn12, Cys14–Ser16, Glu20, Gly36, Leu37, Leu40, Arg66 and Tyr117).

Structural comparison of eukaryotic and prokaryotic LMW-PTPs has revealed that substrate recognition mechanism is different between them, thus, can be exploited for drug design that is more specific toward prokaryotic PTPs than host phosphatases (Lescop et al., 2006). For instance, eukaryotic LMW-PTPs have phosphotyrosine sandwiched between two aromatic rings (Tyr131 and Trp49 in humans and bovine) whereas Wzb has Tyr117 (equivalent to human Tyr131) and doesn’t have Trp49 equivalent. Wzb has shorter α2-β2 loop which marks the difference in substrate recognition. In addition, prokaryotic Wzb recognizes C-terminal tyrosine rich tail of Wzc whereas eukaryotic counterparts recognize a single tyrosine residue edged by charged amino acids.

## Wza: an Outer Membrane Translocon

Wza is an outer membrane translocon that translocates the nascent CPS onto the bacterial surface (Beis et al., 2004; Hagelueken et al., 2009b) (**Figures 8** and **9B**). In fact, recently it has been shown that CPS exits through Wza portal (Nickerson et al., 2014). Knockout of *wza* has been shown to be ineffective in exporting CPS (Nesper et al., 2003; Dong et al., 2006). Wza exists in octameric form (Drummelsmith and Whitfield, 2000; Ford et al., 2009) spanning 140 Å along the periplasmic and outer membrane regions. Acylation at the N-terminal cysteine is important for the octamerization of the protein (Nesper et al., 2003). Octamerization of Wza results in a wider lumen so as to facilitate the surface exportation of CPS and the overall structure of Wza resembles an amphora without a handle (Dong et al., 2006). Bulk of the protein is located in periplasmic space and it also has a novel α-helical transmembrane region (Collins et al., 2007).

There are four domains in the Wza monomer. Upon octamerization, these domains individually form four different rings: I, II, III, and IV. First domain, located in the periplasmic region, comprises of 89–169 residues, which folds into novel anti-parallel β-sandwich with α-helix at one edge constitute ring I. It has an occluded center due to the presence of eight loops. Tyrosine 110 located at the tip of each loop has flexible side chain that is responsible for blocking the pore. Second domain consists of residues 68–84 and 175–252, eight copies of which forms ring II with 25 Å diameter. This domain has a central five-stranded mixed β-sheet with three α-helices on one side. Octamerization of domain three forms ring III with an external diameter of 105 Å. It comprises of residues 46–64 and 255–344. Domain four is the carboxy terminal (345–376 residues) α-helical transmembrane region. This domain sits just above domain three and forms ring IV with internal diameter of 30 Å, which tapers to 17 Å at the open end. Both the central cavity and external surface of first three domains are polar in nature in sync with nature of periplasmic region, while the external surface of α-helical barrel is hydrophobic in nature with tryptophan 350 exposed at the surface (Dong et al., 2006).

Concave surface at the bottom has a conserved polysaccharide export sequence (PES) domain that confers it to be potential site for Wzc–Wza interaction (Collins et al., 2007). It has been found that interaction of Wzc with Wza (Reid and Whitfield, 2005) helps in opening of the closed surface in ring I thus, helping in the transport of nascent polysaccharide through Wza to the cell exterior. However, it has been shown that Wzc doesn’t play any role in the assembly of Wza (Nesper et al., 2003). Defects in export of CPS make the bacterium vulnerable to host immune system. Concomitant to this fact an effective blocker γ-cyclodextrin, has been discovered that not only blocks Wza channel but also enhances complement-mediated cascade (Kong et al., 2013).

## Model Proposed for Group 1 Cps Polymerization and Surface Exportation

**FIGURE 11:**
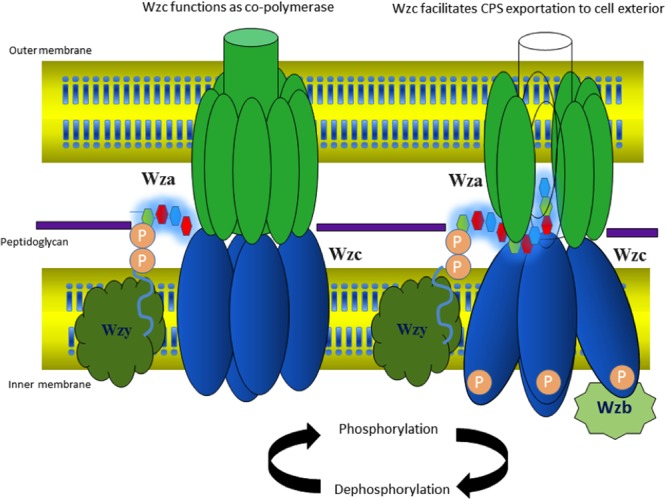
**Model proposed for cyclic copolymerase and CPS exportation activities of Wzc. (Left)** Wzc is in dephosphorylated state with its monomers interacting at the cytoplasmic region and performs the role of copolymerase conjointly with Wzy (colored white). **(Right)** Wzc is in phosphorylated state, wherein the monomers are non-interacting at the cytoplasmic region and participates in exportation of nascent CPS along with Wza. The model is based on existing experimental data on *E. coli* Wza (colored green), Wzb (colored maroon) and Wzc along with Wzc (colored blue) homolog present in polysaccharide exportation system of *S. aureus* (see text for details). Note that the tetrameric state of Wzc is adopted from low-resolution electron microcopy structure of Wzc (Collins et al., 2006).

Various aspects of Group 1 CPS exportation nanomachinery have not been fully understood at the molecular level to provide full mechanistic picture of CPS biosynthesis and surface expression processes. However, existing structural and functional data about the components of CPS exportation machinery provide significant insights about versatility of Wza, Wzc, and Wzb in capsule length regulation and exportation and led us to propose a model. As the tyrosines present in the cytoplasmic region of Wzc can be phosphorylated or dephosphorylated (Doublet et al., 2002), it can regulate the CPS polymerization and exportation activity in a cyclic manner concomitant with dephosphorylation activity of Wzb (Standish and Morona, 2014). Upon dephosphorylation by phosphatase Wzb, Wzc exhibits copolymerase activity conjointly with Wzy by establishing the linkage between the CPS repeating units, thus, helping in CPS chain elongation. During this state, cytoplasmic domains of Wzc multimer are in interactive state (**Figure 11**). On the other hand, phosphorylation of cytoplasmic tyrosines direct cytoplasmic domains to enter into non-interactive state (**Figure 11**), while the periplasmic and transmembrane domains are still in interactive state. This state of Wzc may help in pushing the nascent polysaccharide into Wza channel, thus facilitating polysaccharide exportation. However, the associated conformational changes that these proteins undergo are still unknown. Such cycling between phosphorylated and dephosphorylated state may be needed to couple the actions of this membrane spanning multienzyme complex to carry out CPS biosynthesis and exportation (Olivares-Illana et al., 2008). Further experimentation is needed to understand the missing links to ease the exploitation of the proteins involved in Group 1 CPS exportation for next generation antibiotics.

## Wzi: an Outer Membrane Protein Functioning as a Lection and a Porin

Crystal structure of Wzi indicates that it is an 18-stranded β-barreled outer membrane protein (Bushell et al., 2010, 2013). The barrel has a diameter of ∼36 Å. There are nine loops on extracellular side and 3 amphipathic α-helices and 8 turns at periplasmic end that occlude the pore. The irregular lengths of β strands result in a triangular “notch” at the extracellular side.

**FIGURE 12:**
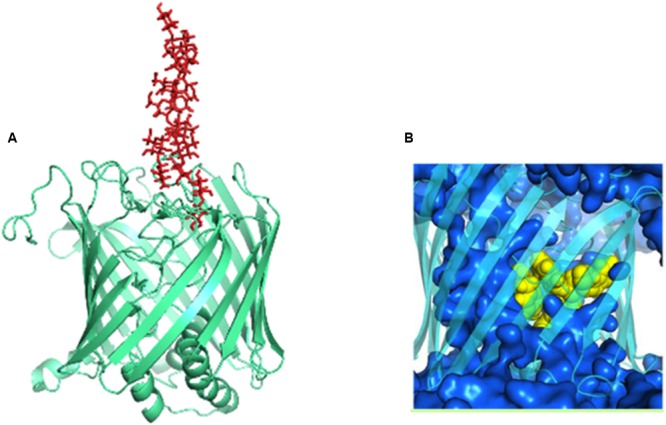
**Dual functions of Wzi. (A)** Lectin function (pale-green colored cartoon) of Wzi (PBD ID: 2YNK) illustrated using K30 (red sticks) modeled structure (Kunduru et al., 2016). **(B)** Water conducting (blue surface) action of Wzi (cyan colored cartoon) (Sachdeva et al., 2016). Residues that direct water conduction across the channels are shown as yellow spheres.

The nascent *K*-antigen binds to Wzi (**Figure 12A**) thus, nucleating its condensation into a dense capsule (Rahn et al., 2003). In fact, insertion mutant of Wzi either forms loosely attached *K*-antigen or the capsule formation is not observed (Rahn et al., 2003). The exact binding site for CPS on Wzi is still unknown. However, similar to sodium galactose cotransporters, an YQF motif is present at the extracellular side of the barrel, suggesting that it may aid in CPS binding (Sachdeva et al., 2016). Molecular dynamics simulations along with structure based phylogeny show that Wzi plays a dual role, as a lectin (sugar binding protein) and a porin (water conducting protein). Porin property (**Figure 12B**) of Wzi may facilitate in keeping the capsule in hydrated condition by acting as an osmoregulator (Sachdeva et al., 2016). Bidirectional water specific porin function of Wzi is justified through its structural similarity with other porins. It has been proposed that the protein might have evolved the lectin property when the bacteria established the self-defense mechanism for its survival.

## Cps Based Vaccine Development

Surface assembled *K*-antigen or CPS is a good vaccine target. The history of CPS based vaccine began in 1920s with the discovery of immunogenicity against CPS in *Streptococcus pneumoniae* (Heidelberger and Avery, 1923, 1924). Consequently, it boosted the discovery of many CPS based vaccines against Gram-negative bacteria such as *Neisseria meningitides, Haemophilus influenza*, and *Salmonella enterica* serovar Typhi (Hutter and Lepenies, 2015). Unlike antibiotics, vaccines prevent the infection. CPS based vaccines are based on the principle of producing antibodies against CPS by vaccinating with an attenuated CPS carrying pathogen. In fact, in *E. coli* many successful attempts have been made in line to develop CPS based vaccines (Mobley and Alteri, 2015). Very recently, a two-stage synergistic ‘vaccination and block’ approach has been successfully used against Group 1 *E. coli* by conjointly targeting LPS (with vaccine) and CPS (with blocker) (Bundle, 2016; Kong et al., 2016). In this approach, a synthetic glycoconjugate that mimics the LPS epitope, which is generally obscured by CPS, is used to generate the antibodies. Subsequently, the LPS epitope is exposed for antibody attack by simply blocking the CPS exporter (Wza) at the extracellular side using γ-cyclodextrin to reduce the CPS exportation. However, poor sequence similarity of Wza among various Gram-negative bacterial strains poses major challenge to develop a universal CPS inhibitor. Water solubility of γ-cyclodextrin is a yet another factor to be addressed.

## Future Perspective

Development of antibiotic resistance through new mechanisms on a regular basis and its ability to transmit between various clinically relevant strains and pathogens is a worrying and challenging scenario. While the number of pathogens exhibiting resistance is increasing day-by-day, the development of next-generation antibacterial drugs to circumvent the existing resistance mechanism is in very slow phase. As suggested by O’Neill (2016) in AMR review, finding new antibiotic resistance mechanisms that can be targeted, judicious use of antibiotics in human, veterinary, and aquaculture medicine, eliminating the use of antibiotics in agriculture and following hygienic practice through public awareness may slowdown the evolution of AMR, but, will not eradicate it. Though synergetic use of different antibiotics can be useful, use of antivirulence drugs/vaccines would help in reducing the selection pressure on the bacteria.

Biogenesis of cell envelope is a valuable target for antivirulence drugs in *E. coli*. Three mechanisms can be hampered in this regard: biosynthesis, export and surface assembly of polysaccharides. In *E. coli* Wzy-dependent Group 1 *K*-antigen pathway, capsule biosynthesis and export can be prevented by blocking the function of regulatory proteins Wzc and Wzb as they together alter the thickness of CPS at different stages of the infection, making them viable drug targets (Ericsson et al., 2012). In fact, a recent study shows that knockout of *wza, wzc*, and *wzb* offers resistance to macrolide in capsule independent manner (Botros et al., 2015). However, a single knock out of *wzb* doesn’t exhibit resistance to the macrolide erythromycin (Rana et al., 2016). As acknowledged by the authors themselves more systematic investigation on effect of the single and combination of double knockouts on capsule surface expression are required against various antibiotics against various *E. coli* strains to derive a solid conclusion on this aspect.

**FIGURE 13:**
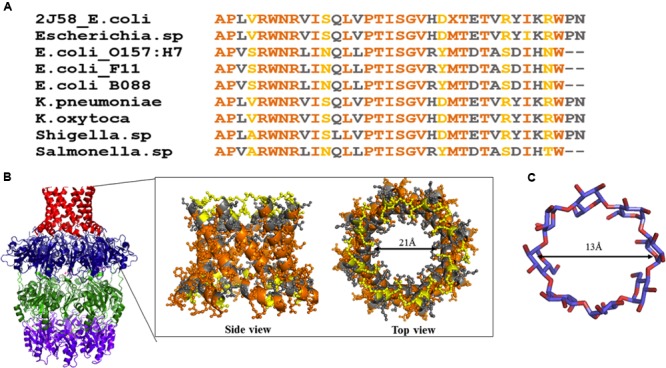
**Sequence analysis of α helical barrel of Wza. (A)** Sequence alignment of Wza α helical sequences between various *E. coli* and Gram-negative bacterial strains. Note that multiple sequence alignment was done using MEGA 7.0 (Kumar et al., 2016). **(B)** Conserved and mutated residues of alpha helical barrel of Wza mapped onto Wza structure (boxed region). Note that while sequence identity and similarity are colored orange and gray respectively, the mutated residues are colored yellow. High sequence conservation of amino acids at the protein - membrane interface and divergence in the sequence at the extracellular side can be seen. **(C)** Structure of gamma cyclodextrin that is successfully shown to block the binding of K30 CPS to Wza.

Wza (an outer membrane translocon) and Wzi (an outer membrane lectin and porin) are the most suitable antivirulence targets as they are surface exposed. Both the proteins are essential for the production of a normal physiological capsule. It has been shown that knock out of *wza* offers resistance to erythromycin in capsule independent manner (Rana et al., 2016). Wza can be blocked using carbohydrate blockers such as γ-cyclodextrin (Kong et al., 2013). However, the membrane spanning alpha helical barrel of Wza sequence is highly diverse at the extracellular side even among *E. coli* strains (**Figure 13A**). This poses a major challenge in developing a universal drug molecule against Gram-negative bacteria that uses Wzy-dependent CPS exportation pathway, as the residues exposed to extracellular side are crucial in dictating the binding specificity with drug molecules. This is true despite the fact that Wza blocker like γ-cyclodextrin can easily fit into the Wza channel: ∼13Å diameter (former) versus 21 Å (latter) (**Figures 13B,C**). It is possible that chemical modification of γ-cyclodextrin molecule would help to solve the issue, at least to treat pathogenic *E. coli*.

Wzi can be ambushed in three different ways. Firstly, by targeting its lectin property, and identifying the binding site of *K*-antigen on Wzi is of paramount importance to hamper the capsule formation. Crystal structure of Wzi (Bushell et al., 2013) along with EK3D (Kunduru et al., 2016), a manually curated repository of modeled *E. coli K*-antigen structures can be exploited for this strategy. Secondly, disrupting the anchorage of Wzi onto the membrane may in turn prevent the capsule formation. Thirdly, altering water diffusion property of Wzi may result in cell rupturing, thus killing the bacterium. Yet another antivirulence strategy is to develop cell envelope based vaccines to treat the infections. In fact, so far there is no Food and Drug Administration US (FDA) approved vaccines available against *E. coli* infections and only two vaccines are in Phase I clinical trial (O’Neill, 2016). Conjoint use of antibiotics along with vaccines may reduce the proliferation of infectious bacteria. Nonetheless, certain issues pose challenges in developing vaccines against surface polysaccharides. For instance, presence of a molecular mimicry of a *K*-antigen in the host reduces the production of antibodies against specific *K*-antigen, resulting in a vaccine with less immunogenicity. Due to the strain diversity, designing a surface polysaccharide-based vaccine that can be effective against infections caused by all serotypes is extremely challenging.

## Author Contributions

TR formulated the outline of the manuscript. SS, RP, and KS collected the data. SS, RP, KS, and TR wrote the manuscript.

## Conflict of Interest Statement

The authors declare that the research was conducted in the absence of any commercial or financial relationships that could be construed as a potential conflict of interest.

## Footnotes

1 https://www.cdc.gov/ecoli/2016/o157-02-16/

2 http://www.fda.gov/Food/RecallsOutbreaksEmergencies/Outbreaks/ucm487651.htm
